# Informatics Competency and Technology Self-Efficacy Levels Among Undergraduate Nursing Students: Cross-Sectional Study

**DOI:** 10.2196/85283

**Published:** 2026-06-08

**Authors:** Nader Alnomasy, Habib Alrashedi, Sharifah Alsayed, Petelyne Pangket, Razan Alsayed

**Affiliations:** 1 Medical Surgical Department, College of Nursing, University of Ha'il, PO Box 2240, Hail, 55349, Saudi Arabia, 966 0532476596; 2 Medical Surgical Department, College of Nursing, King Saud bin Abdulaziz University for Health Sciences, Jeddah, Saudi Arabia; 3 Medical Surgical Department, College of Nursing, Taif University, Taif, Mecca Region, Saudi Arabia; 4 Nursing Education Department, King Fahd Armed Forces Hospital, Jeddah, Mecca Region, Saudi Arabia

**Keywords:** informatics competency, digital health, nursing education, self-efficacy, electronic health records

## Abstract

**Background:**

The Saudi Arabian health care sector is transforming under Vision 2030 with the goal of digitizing its services. This necessitates a digitally prepared nursing workforce; however, there is evidence suggesting that nursing students have limited informatics competency and that these skills are minimally covered in their training programs.

**Objective:**

This study measured the baseline informatics competency and technology self-efficacy of undergraduate nursing students at the University of Hail, Saudi Arabia.

**Methods:**

Using a descriptive cross-sectional design, data were collected from 243 undergraduate nursing students at the University of Hail through an online survey. The survey measured demographics, informatics competency (Canadian Nurse Informatics Competency Assessment Scale), and digital technology self-efficacy. Data analysis used descriptive statistics, 2-tailed *t* tests, ANOVA, and hierarchical regression analysis.

**Results:**

Students reported a moderate level of informatics competency, with a mean Canadian Nurse Informatics Competency Assessment Scale item score of 2.57 (SD 0.84) on a scale from 1 to 4. They also showed moderate to high self-efficacy for digital technology, with a mean item score of 2.7 (SD 0.56) out of 4. Informatics competency scores were substantially higher in students with prior informatics training and frequent electronic health record exposure. In the underpowered exploratory hierarchical regression model, self-efficacy for digital technology showed a small positive association with informatics competency that approached statistical significance; however, the overall model was not statistically significant and explained only a limited proportion of the variance.

**Conclusions:**

This single-site study of undergraduate nursing students at the University of Hail found moderate self-reported informatics competency despite higher digital technology self-efficacy, indicating a need for strengthened informatics education to support the Saudi Vision 2030. While underpowered, exploratory analyses and self-report data mean that these institution-specific findings should be interpreted cautiously and used primarily to guide future multisite and mixed methods research.

## Introduction

The digital health revolution continues to transform health care delivery and organizational structures. Advances in artificial intelligence (AI), telemedicine, and electronic health records (EHRs) now support safer, more efficient, and data-driven care [1,2]. Consequently, preparing a globally competent health informatics workforce is essential for implementing these innovations and enhancing clinical outcomes [3,4].

Nurses comprise the largest portion of the health care workforce and are in a pivotal position to integrate technology into direct patient care. Informatics competency, the integration of computers to manage and communicate data [3], is a key skill that nurses must develop. Equally important is technology self-efficacy, or nurses’ confidence in using digital tools in clinical and educational settings [5]. Despite the wide recognition of their importance, the inclusion of nursing informatics in undergraduate curricula varies considerably worldwide. Studies from North America, Europe, Africa, and the Asia-Pacific region have shown significant differences in course content, learning opportunities, and assessment methods used to measure informatics competency among nursing students [6-8].

International initiatives such as Technology Informatics Guiding Education Reform and the Canadian Nurse Informatics Competency Assessment Scale (C-NICAS) promote the formal integration of informatics into nursing education. However, their adoption varies widely across different programs and countries. This study used the C-NICAS as an assessment tool and conceptual framework to identify key informatics competency domains among undergraduate nursing students. Despite the global use of such frameworks, empirical studies have shown that students and new graduates consistently self-report only basic to intermediate proficiency, often lacking hands-on experience with clinical information systems. This highlights the persistent gap between curriculum design and real-world practice [4].

As part of Vision 2030, Saudi Arabia has launched a nationwide digital health initiative to modernize health care using digital tools, telemedicine, and AI [9-11]. Initiatives such as the Healthcare Sector Transformation Program aim to transform service delivery, improve accessibility, and enhance the quality and efficiency of care, reflecting a national focus on developing a data-driven, patient-centered workforce. However, studies have shown that Saudi nursing students and practicing registered nurses have lower informatics competency than their international counterparts, particularly those in countries with more advanced digital technologies and infrastructure [12-15]. Despite significant reform efforts, the integration of informatics into Saudi nursing education has not yet reached the level observed in many other high-income countries, indicating the need for targeted policy and curriculum intervention.

Although many global models advocate for the integration of informatics into nursing education, very little research has addressed this topic in Saudi Arabia. Few studies have investigated how prepared nursing students are for digital health roles or how their informatics competency relates to their confidence with technology, both of which are important factors for successfully joining today’s digital health care workforce. These research gaps need to be filled so that evidence can guide faculty training and improve nursing curricula, especially given the Vision 2030 focus on digital health. This study measured the baseline informatics competency and digital readiness of nursing students at the University of Hail, Saudi Arabia. The goal was to provide local data that address a known gap in preparing Saudi Arabia’s future nurses to work in digitally transformed health care settings.

## Methods

### Study Design

This study adopted a descriptive cross-sectional design to assess perceived informatics competency and digital technology self-efficacy among undergraduate nursing students using an online self-assessment survey.

### Study Population and Sampling

Voluntary participation in an electronic survey allowed for data collection from undergraduate nursing students at a university in Hail, covering students from the first to fourth years (levels 1‐4) of the Bachelor of Nursing program. The target population was all undergraduate nursing students enrolled in the Bachelor of Nursing program at the University of Hail during the 2024‐2025 academic year (levels 1‐4 and interns). The source population comprised all students with an active institutional email address on the official program mailing list at the time of data collection. Therefore, the accessible sampling frame consisted of this official mailing list of currently enrolled undergraduate nursing students, which was used to distribute a single open invitation to participate in the study.

Students were eligible if they (1) were currently enrolled as undergraduate nursing students in levels 1 to 4 at the University of Hail, (2) were able to read and understand English (the language of instruction), and (3) had an active institutional email account. Students on academic suspension, on long-term leave, or who had withdrawn from the program at the time of data collection were excluded from the study.

A convenience sampling approach was used in which all eligible students from the source population were invited to participate. This strategy was practical, cost-effective, and enabled the coverage of the broadest possible segment of the accessible student population. In this context, convenience sampling reflects all students who chose to respond to the open email invitation rather than a preselected subgroup. All invitations were distributed via the official undergraduate nursing program’s email list. Because the list is updated dynamically, the exact number of invitations sent could not be confirmed, but it approximately matched the total number of eligible students. The survey was conducted using Google Forms. A total of 260 students started the survey, of whom 93.5% (243/260) completed it and were included in the analysis. In total, 6.5% (n=17) of the responses were excluded due to substantial missing data (more than 20% of the items were unanswered). Because the total number of active student email accounts at the time of the survey could not be verified, the exact response rate could not be calculated. However, program records indicated that the mailing list approximately matched the total number of enrolled and eligible students.

For inferential analyses involving year level, only students in levels 1 to 4 were included, excluding interns because their internship year is structurally distinct from the numbered academic levels. To reduce potential selection bias, all eligible undergraduate nursing students on the institutional mailing list were invited to participate using the recruitment protocol described in the Data Collection section.

### Study Size

A priori power analysis indicated that at least 315 respondents were required to achieve 80% power at a 95% confidence level for medium effects in ANOVA, 2-tailed *t* tests, and regression analyses. Therefore, the final analytic sample of 243 respondents was underpowered for confirmatory inferential analyses but was considered adequate for descriptive and exploratory correlational analyses.

### Instrument

The questionnaire was divided into 3 sections to capture comprehensive participant information. The first section collected general demographic data, including age, sex, academic year, prior experience with electronic medical records, and self-assessed computer proficiency. These variables provided a contextual background for interpreting the findings in the subsequent sections.

The second section assessed participants’ informatics competency using the 21-item C-NICAS version 2 with permission from Kleib and Nagle [3]. This student-focused instrument has been adapted and validated for undergraduate nursing students across multiple international contexts, supporting its suitability for this population and enabling cross-study comparison. Originally developed and psychometrically validated among registered nurses in Alberta, Canada, the scale has also undergone cross-cultural validation, supporting its use in international benchmarking.

The C-NICAS includes 4 subscales: foundational information and communications technology (ICT) skills, information and knowledge management, professional responsibility and accountability, and use of ICTs in the delivery of care. These domains align with the core competencies relevant to the digital health ecosystem in Saudi Arabia. The instrument was administered in English, consistent with the language of instruction at the study site. Participants self-reported their competency using a 4-point Likert scale ranging from 1 (“not competent”) to 4 (“very competent”). The total scores ranged from 21 to 84 and were categorized into 4 levels: not competent (21-41), somewhat competent (42-62), competent (63-83), and very competent (84). The scale demonstrated strong internal consistency (Cronbach α=0.93 overall and 0.74‐0.89 for the subscales).

The final section measured digital technology self-efficacy using a revised 17-item version of the questionnaire by Hughes [16]. This scale complements the C-NICAS by assessing students’ confidence in their ability to use digital technology. Responses were recorded on a 4-point Likert scale ranging from 1 (“strongly disagree”) to 4 (“strongly agree”). In total, 12 items were reverse coded, and 5 items (1, 7, 8, 11, and 16) were positively coded. The mean scores ranged from 1 to 4, with higher scores indicating greater self-efficacy. The scale demonstrated strong reliability, with the Cronbach α exceeding 0.90 in both the original study and the sample in this study.

Both informatics competency and digital technology self-efficacy were assessed using self-report measures. Although both instruments have strong psychometric properties, self-reported data may be subject to bias and inaccurate self-assessment, potentially leading to over- or underestimation of actual competency and confidence levels.

### Data Collection

An ethical recruitment plan was developed. Nursing students were invited through email, which contained an invitation letter, a survey link, an information sheet, and a consent statement. The invitation highlighted the purpose of the research, the importance of the study, and the possible implications of the study for nursing informatics research.

Reminder emails using a predetermined protocol were sent to encourage response; the first reminder was sent 24 to 48 hours after the initial email, followed by another one 3 to 7 days later, with a total of 3 to 5 reminders sent throughout the data collection period. Google Forms was used for the survey because of its accessibility and reasonable data storage capacity.

### Statistical Analysis

Data analysis was performed using SPSS (version 29.0; IBM Corp) and the Python software (Python Software Foundation). Item-level missing data were minimal (<5% for all variables). Continuous items with less than 5% of missing data were imputed using item-specific means and categorical variables using the mode. Given the low proportion of missing data, this approach was unlikely to bias the descriptive estimates, although it may have slightly attenuated the variance in the inferential analyses.

Statistical assumptions were assessed prior to the inferential analyses. Descriptive statistics, including measures of central tendency (mean, median, and mode) and variability (SD), were calculated for key variables, such as C-NICAS and digital technology self-efficacy scores. Inferential analyses included Pearson correlation to examine relationships among variables, independent-sample *t* tests, and one-way ANOVA to assess group differences.

Hierarchical multiple regression was conducted using 4 models to identify the predictors of informatics competency. Potential confounders (age, sex, academic level, prior informatics training, and previous EHR exposure) were included; however, given the underpowered sample and limited variables, residual and unmeasured confounding (eg, informal IT training or personal interest in technology) cannot be excluded.

The significance level was set at an α value of .05. Effect sizes were interpreted using the Cohen standards for correlations (small=0.10; medium=0.30; large=0.50), and *F* change statistics were used to assess changes in *R*^2^ values across regression models. As the final sample was below the a priori target (N=243 vs 315), all inferential analyses should be considered exploratory and hypothesis generating rather than confirmatory.

### Ethical Considerations

Institutional review board approval from the University of Hail (H-2024-437) was obtained prior to data collection. Participation was entirely voluntary and no financial compensation was provided to the participants. All procedures were conducted in accordance with the ethical standards of the Research Ethics Committee and the 1964 Declaration of Helsinki and its subsequent amendments.

Informed consent was obtained from all students prior to accessing the online survey and questionnaire. Participation was entirely voluntary, and students had the right to withdraw at any time without adverse academic consequences. To preserve participants’ anonymity, no personal identifiers (such as names or email addresses) were collected through Google Forms, and the researchers did not link any responses to individual participants. The dataset was stored on a secure, password-protected server, and access was limited to the research team members.

This study posed an extremely low risk as it involved noninvasive procedures and presented no more than minimal risk to the participants. The participants’ normal coursework was not affected, and it was clearly stated that their decision to participate or not would have no impact on their academic status or their relationship with faculty members.

## Results

Table 1 presents the study’s 243 undergraduate nursing student participants from Saudi Arabia, with most being young adults aged 21 to 25 years (n=116, 47.7%) and only 7% (n=17) aged 26 years and above. The sample was predominantly female (n=159, 65.4%). Students were distributed across academic levels, with interns accounting for the largest group (n=63, 25.9%) and year level 8 students representing the smallest group (n=13, 5.3%). The participants were nearly equally divided between those with informatics training (n=124, 51%) and those without (n=119, 49%). Comfort with digital technology varied, with 41.2% (n=100) expressing discomfort and 22.2% (n=54) remaining neutral on the subject. EHR experience ranged from 40.3% (n=98) having occasional exposure to 29.6% (n=72) having no prior experience with EHRs. Most participants demonstrated good technology accessibility, with 82.3% (n=200) using technology daily for educational purposes and 65% (n=158) reporting excellent home internet access. Regarding career intentions, most (n=130, 53.5%) planned to pursue clinical care, whereas 13.6% (n=33) remained undecided about their future nursing career paths. A comparison between respondents and nonrespondents was not possible because identifiable sampling frame data were unavailable; therefore, some nonresponse bias cannot be excluded.

**Table 1.** Demographic characteristics (N=243).

Category	Values, n (%)
Age (years)	
≤20	110 (45.3)
21-25	116 (47.7)
≥26	17 (7)
Sex	
Male	84 (34.6)
Female	159 (65.4)
Year level	
First	69 (28.4)
Second	72 (29.6)
Third	39 (16)
Fourth	63 (25.9)
Have you previously completed any informatics-related training or coursework?	
No	119 (49)
Yes	124 (51)
Rate your comfort level with digital technology (eg, computers, smartphones, and EHRs^a^)	
Very uncomfortable	47 (19.3)
Uncomfortable	53 (21.8)
Neutral	54 (22.2)
Comfortable	89 (36.6)
Previous exposure to EHRs	
No experience	72 (29.6)
Yes, occasionally (monthly or less)	98 (40.3)
Yes, frequently (weekly or daily)	73 (30)
Do you own or regularly have access to a personal computer or laptop?	
No	49 (20.2)
Yes	194 (79.8)
How often do you use technology (smartphones, tablets, or computers) for educational purposes?	
Monthly	11 (4.5)
Weekly	32 (13.2)
Daily	200 (82.3)
Internet accessibility at home	
Fair	19 (7.8)
Good	66 (27.2)
Excellent	158 (65)
Intended future nursing career area	
Undecided	33 (13.6)
Administration or leadership role	14 (5.8)
Academic or teaching role	35 (14.4)
Nursing informatics	14 (5.8)
Public health or community nursing	17 (7)
Clinical care (hospital or clinical setting)	130 (53.5)

a EHR: electronic health record.

Table 2 presents the self-reported C-NICAS item scores from the 243 undergraduate nursing students (mean 2.57, SD 0.84 on a scale from 1-4); the median was 2.50 (2.00-3.00). Regarding the total number of C-NICAS scores, the largest percentage of students (n=116, 47.7%) achieved a score of “somewhat competent”; 21.4% (n=52), or nearly 1 in 5, achieved a score of “not competent”; 23.9% (n=58) achieved a score of “competent”; and only 7% (n=17) achieved a score of “very competent.” When reviewing the subscales, the “foundational ICT skills” subscale had the highest average C-NICAS item score (2.62, SD 0.93), followed by the “use of ICT in the delivery of patient care” (2.60, SD 0.85) and “professional and regulatory accountability” (2.56, SD 0.88) subscales, whereas the “information and knowledge management” subscale had the lowest average C-NICAS item score (2.51, SD 0.86). Most students reported that they rated themselves as somewhat competent on 3 out of the 4 subscales (n=75, 30.9%‐n=106, 43.6%), and most students who evaluated themselves as not competent did so on the “foundational ICT skills” subscale (n=58, 23.9%) and least frequently in the “use of ICT in the delivery of patient care” subscale (n=43, 17.7%). Overall, students self-reported moderate levels of informatics competency; however, many rated themselves as only somewhat competent in practically all areas of informatics competency.

**Table 2.** Nursing students’ perceptions of informatics competency (Canadian Nurse Informatics Competency Assessment Scale [C-NICAS] mean item scores; N=243).

Scale and subscale	Item score (1-4), mean (SD)	Item score (1-4), median (IQR)	Range	Not competent, n (%)	Somewhat competent, n (%)	Competent, n (%)	Very competent, n (%)
NI^a^ competency (overall C-NICAS)	2.57 (0.84)	2.50 (2.01–3.10)	1-4	52 (21.4)	116 (47.7)	58 (23.9)	17 (7)
Foundational ICT^b^ skills	2.62 (0.93)	2.66 (2.00–3.33)	1-4	58 (23.9)	75 (30.9)	78 (32.1)	32 (13.2)
Information and technology management	2.51 (0.86)	2.50 (2.00–3.00)	1-4	57 (23.5)	103 (42.4)	57 (23.5)	26(10.7)
Professional and regulatory accountability	2.56 (0.88)	2.50 (2.00–3.17)	1-4	52 (21.4)	102 (42)	58 (23.9)	31 (12.8)
Use of ICT in the delivery of patient care	2.60 (0.85)	2.66 (2.00–3.17)	1-4	43 (17.7)	106 (43.6)	65 (26.7)	29 (11.9)

a NI: nursing informatics

b ICT: information and communications technology.

The mean self-efficacy score for the use of digital technologies was 2.7 (SD 0.56) on a scale from 1 to 4. This indicates that, as a group, the nursing students reported a moderate to high level of confidence in using digital technologies. Because the mean score of 2.7 is above the scale midpoint, the students’ digital technology self-efficacy can be considered strong.

Table 3 presents the data on informatics competency and digital technology self-efficacy among Saudi nursing students. There were no statistically significant differences in the C-NICAS total scores by year level for students in levels 3 to 8 (*P*=.48) or in the digital technology self-efficacy mean item scores (*P*=.87). The mean C-NICAS total scores ranged from 48.3 (SD 15.6) in level 3 to 55.4 (SD 17.9) in level 7, whereas the digital technology self-efficacy mean item scores ranged from 2.5 (SD 0.5) to 2.7 (SD 0.2) on a scale from 1 to 4. Exploratory *t* test analysis showed that students with prior informatics training had higher mean C-NICAS total scores (55.0, SD 15.8) than those without such training (50.6, SD 15.2; *P*=.005), whereas their mean digital technology self-efficacy item scores were similar (2.6, SD 0.4 vs 2.5, SD 0.5; *P*=.53). Exploratory ANOVA further indicated that students with no EHR experience had the lowest mean C-NICAS total scores (48.4, SD 16.1) compared with higher scores among those with occasional (53.4, SD 17.2) or frequent (54.9, SD 13.4) EHR use (*P*=.002); in contrast, digital technology self-efficacy mean item scores remained relatively stable across groups with EHR use (2.5, SD 0.4 to 2.6, SD 0.4; *P*=.40).

**Table 3.** Overall competency and self-efficacy levels by year level, prior informatics training, and routine electronic health record (EHR) use.

Variable	C-NICAS^a^ total score (21-84), mean (SD)	*P* value	DT-SE^b^ item score (1-4), mean (SD)	*P* value
Year level		.48		.87
3	48.3 (15.6)		2.6 (0.4)	
4	53.1 (16.2)		2.6 (0.4)	
5	53.5 (16.7)		2.5 (0.5)	
6	53.2 (14.3)		2.6 (0.5)	
7	55.4 (17.9)		2.7 (0.2)	
8	53.7 (15.7)		2.6 (0.4)	
Informatics training		.005		.53
Yes	55.0 (15.8)		2.6 (0.4)	
No	50.6 (15.2)		2.5 (0.5)	
EHR use		.002		.40
No experience	48.4 (16.1)		2.6 (0.4)	
Occasional	53.4 (17.2)		2.5 (0.4)	
Frequent	54.9 (13.4)		2.6 (0.4)	

a C-NICAS: Canadian Nurse Informatics Competency Assessment Scale.

b DT-SE: digital technology self-efficacy.

Table 4 illustrates the preliminary correlational findings showing a positive association between digital technology self-efficacy and 3 of the informatics competency subscales. The strongest correlation was with foundational ICT skills (*r*=0.181; *P*=.002), followed by professional responsibility and accountability (*r*=0.157; *P*=.002) and use of ICT in care delivery (*r*=0.113; *P*=.02). However, the strongest correlations were between the competency subscales themselves, indicating a good level of internal connection. Specifically, the strongest correlation was between professional accountability and use of ICT in care (*r*=0.794; *P*=.001), followed closely by the correlation between foundational ICT skills and professional accountability (*r*=0.769; *P*=.001) and between foundational ICT skills and use of ICT in care (*r*=0.765; *P*=.001).

**Table 4.** Correlation between competency and self-efficacy.

	DT-SE^a^	Foundational ICT^b^ skills	Professional and regulatory accountability	Use of ICT in the delivery of patient care
DT-SE				
*r*	1	0.181	0.157	0.113
*P* value	—^c^	.002	.002	.02
Foundational ICT skills				
*r*	0.181	1	0.769	0.765
*P* value	.002	—	.001	.001
Professional and regulatory accountability				
*r*	0.157	0.769	1	0.794
*P* value	.002	.001	—	.001
Use of ICT in the delivery of patient care				
*r*	0.113	0.765	0.794	1
*P* value	.02	.001	.001	—

a DT-SE: digital technology self-efficacy.

b ICT: information and communications technology.

c Not applicable.

Table 5 presents the outcomes of an exploratory hierarchical multiple regression analysis examining potential predictors of informatics competency measured using the C-NICAS score. In model 1, age and sex were entered, yielding an *R* value of 0.013 and an adjusted *R*^2^ value of 0.005 (*F*_2,243_=1.611; *P*=.20), with no significant predictors. In model 2, year level (fourth-eighth) and internship status were added, resulting in an *R* value of 0.031 and an adjusted *R*^2^ value of −0.002 (*F*_8,237_=0.950; *P*=.48), and again, no significant predictors emerged. In model 3, prior EHR experience and informatics training were added, yielding an *R* value of 0.054 and an adjusted *R*^2^ value of 0.013 (*F*_10,235_=1.330; *P*=.22), with no variables reaching statistical significance. In the final model (model 4), the mean digital technology self-efficacy score was included, resulting in an *R* value of 0.069 and an adjusted *R*^2^ value of 0.022 (*F*_11,234_=1.577; *P*=.11). Digital technology self-efficacy showed at most a weak, borderline association with informatics competency (*B*=4.34; *P*=.05), accounting for only a negligible proportion of the variance; thus, it cannot be considered a robust predictor in this sample. Given the modest effect size, nonsignificant overall model, and sample size (N=243) falling below the a priori target (N=315), these regression results should be interpreted with caution and viewed as exploratory and hypothesis generating rather than confirmatory. Overall, the hierarchical models explained only a small proportion of the variance in informatics competency scores.

**Table 5.** Hierarchical multiple regression models predicting informatics competency^a^.

Model	Variables entered	*R* ^2^	Adjusted *R*^2^	*F* test (*df*)	*P* value	Significant predictors (final model)
1	Age and sex	0.013	0.005	1.611 (2, 243)	.20	None
2	+ year level (fourth-eighth) and internship	0.031	−0.002	0.950 (8, 237)	.48	None
3	+ EHR^b^ exposure and informatics training	0.054	0.013	1.330 (10, 235)	.22	None
4	+ mean digital technology self-efficacy score	0.069	0.022	1.577 (11, 234)	.11	Digital technology self-efficacy (*B*=4.34; *P*=.05)

a None of the hierarchical regression models reached conventional statistical significance for the overall *F* test, and the explained variance was small (maximum adjusted *R*2=0.022); therefore, these findings, including the trend-level association for digital technology self-efficacy, should be interpreted as exploratory and preliminary.

b EHR: electronic health record.

## Discussion

### Principal Findings

#### Overview

This study assessed the baseline informatics competency of undergraduate nursing students at a single institution in Hail, Saudi Arabia, based on self-reported informatics competency scores and revealed a moderate level of competency. Most students rated themselves as somewhat competent. These results suggest that current competency levels may be insufficient to fully support the digital health priorities articulated in the Saudi Vision 2030, indicating the need to further strengthen informatics education and experiential exposure to digital health systems. In addition, this study found that students with previous informatics education had notably higher C-NICAS scores than those without.

#### Informatics Competency Levels

A moderate level of informatics competency, particularly lower scores in the “information and knowledge management” and “professional responsibility and accountability” subscales, suggests that students may have basic ICT and documentation abilities but lack proficiency in the critical appraisal and interpretation of digital health data, application of ethical and regulatory standards, and more advanced use of information to support clinical decision-making. These deficits are likely related to limited systematically integrated informatics content within undergraduate curricula and restricted, structured exposure to digital health systems, particularly EHRs.

Several studies in Saudi Arabia have reported that nursing students possess basic informatics knowledge but do not consistently achieve high competency [17,18]. The reported deficits are greatest in clinical informatics and applied computer skills, highlighting the gap between knowledge and practice [17,18]. Students proficient in everyday digital technologies often struggle to transfer these abilities to clinical settings that require specific informatics skills [18].

Other studies have reinforced the distinction between digital literacy and informatics competency [19,20]. Nursing students may report moderate or high digital health literacy; however, literacy alone does not ensure competency in critical appraisal, data interpretation, or safe clinical application [19]. Although there have been initiatives to integrate digital skills into the curriculum, the lack of rich experiential learning limits students’ progression to competent or advanced informatics levels [20]. Considering these findings and the C-NICAS results in our study, Saudi nursing programs should substantially expand informatics content across all years of study. The lowest mean scores in the “information and knowledge management” subscale underscore the need for modules that teach students to manage, retrieve, and critically appraise health information from electronic databases. These competencies can be addressed through dedicated courses and by embedding informatics objectives into existing theory and clinical units, consistent with recent international recommendations [21].

Experiential learning should include structured, practice-focused engagement with digital health systems, particularly EHRs, as students with previous EHR exposure in this study demonstrated higher competency. This aligns with evidence that greater EHR integration is associated with improved informatics knowledge and skills [22,23]. Targeted faculty development is also needed so that educators can confidently model and assess informatics competency in both classroom and clinical settings.

Academic-practice partnerships that provide students with supervised access to real-time informatics environments can help bridge the gap between classroom learning and clinical practice. Aligning these curricular changes with the Saudi Vision 2030 health strategy is essential for building a digitally competent nursing workforce capable of supporting high-quality, safe care across the kingdom [9,23,24].

#### Digital Technology Self-Efficacy

The moderately high digital technology self-efficacy found in this study likely stems from students’ heavy use of digital tools, widespread internet access, and regular use of online resources. Although digital technology self-efficacy showed a small positive correlation with informatics competency, the hierarchical regression model was not statistically significant, explaining only 2% of the variance. Consequently, digital technology self-efficacy was not a strong predictor of informatics competency in this study. These results should be viewed as hypothesis generating, suggesting a weak association that requires confirmation in larger samples. Instead of making causal claims, the focus remained on robust descriptive and bivariate findings such as differences related to prior informatics training and EHR exposure.

A trend toward increased willingness to use digital technologies for educational and clinical purposes is evident among frequent users, consistent with the findings of another study [25]. Recent studies indicate that nursing students generally show acceptable preparedness for AI-based health care and a positive disposition toward digital health tools [14,25,26]. In Saudi Arabia, nursing students typically view e-learning favorably and are proficient in technology-rich environments, particularly in blended learning contexts [14]. Prior experience and digital literacy remain key indicators of self-efficacy and preparedness in undergraduate nursing education [26].

Nevertheless, digital technology self-efficacy varies considerably [14,27]. Many students remain uncomfortable with digital technologies, especially when transitioning to clinical settings [27]. Furthermore, some are dissatisfied with e-learning due to a lack of in-person interaction and support [14]. Technical difficulties, inconsistent design, and inadequate training can also decrease confidence and satisfaction [14].

These findings suggest that access and exposure are insufficient. Students face obstacles in applying personal technological knowledge to professional contexts and require more support [14,26]. Digital technology self-efficacy can be strengthened by providing consistent hands-on digital literacy education, individualized assistance, and cooperative blended learning environments. Nursing programs should also build strong technological foundations and systematically collect student feedback to improve these environments [14,26]. This approach will help students develop the confidence and skills necessary to use digital tools effectively in both study and practice.

#### Role of EHR Experience

Early experience with EHRs significantly influences nursing students’ informatics competency. There was a positive relationship between EHR experience and informatics competency: students who reported frequent or occasional hands-on use had higher competency scores than those with little or no experience. Practicing informatics in real time enhances the translation of abstract knowledge into practical applications, and providing students with authentic EHR experiences contributes to their clinical preparation [28]. Exposure to educational EHR platforms requires technical proficiency and has broader implications for student learning.

Institutions emphasizing EHR use can expect improved student performance in informatics assessments and application skills, resulting in graduates who are better prepared for practice [22]. Structured exposure to EHR systems may also enhance students’ documentation skills, clinical reasoning, and compliance with professional and regulatory requirements. As the demand for advanced informatics skills increases, all students will need structured and equitable access to educational EHR systems [28].

Digital literacy and informatics competency gaps are most apparent when students transition from classrooms to clinical settings. Many students who do not receive instruction on EHR use feel unprepared to document clinical data or perform other informatics tasks [28]. Furthermore, obstacles during clinical rotations are multifaceted, including limited EHR access, unclear learning expectations, and variable guidance from preceptors [28].

Similar concerns exist in the Saudi context, where inequitable student access to EHRs at clinical sites has been identified [15]. Nursing programs should provide comprehensive longitudinal training programs that address EHR use. Furthermore, programs should incorporate simulated or laboratory-based experiences that replicate authentic informatics tasks such as data retrieval, clinical decision-making, and regulatory documentation [21].

Finally, partnering with health care organizations can ensure that students have supervised EHR access and clear learning objectives during clinical rotations [28]. These strategies help bridge the gap between basic digital literacy and professional informatics competency, enhancing readiness for real-world practice, increasing patient safety, and creating long-term career opportunities in the digital health care field.

### Implications

These results have significant implications for nursing education and policy in Saudi Arabia. Considering the moderate level of informatics competency, curricular reform is needed to align nursing students with the digital elements of Vision 2030. This can be achieved not only by viewing faculty as presenters of subject matter but also by emphasizing authentic learning experiences. This could be further aided by incorporating a specific informatics module or student engagement activities that rely on EHRs and digital health technologies, which have demonstrated successful clinical experiences in American and Canadian programs. Finally, it is of utmost importance to formalize partnerships between universities and practice settings to ensure that students have authentic experience with real-time informatics in practice settings. These initiatives could yield a digitally competent workforce that can use technology in patient care and contribute to health initiatives in the future.

### Conclusions

This single-site survey found that undergraduate nursing students at the University of Hail showed noticeable gaps in informatics competency even though many reported moderate to high confidence in using digital technologies. This highlights the need to revisit the current curriculum considering Saudi Vision 2030 and its emphasis on digital health. The findings also suggest that being comfortable with everyday technology does not necessarily mean that students are prepared with the informatics skills required in clinical settings.

Students with prior exposure to informatics content or experience with EHRs generally demonstrated better competency levels. However, the study sample was smaller than originally planned, and the statistical analyses were not sufficiently powered. Therefore, any observed relationship, including the weak link between digital technology self-efficacy and informatics competency, should be interpreted with caution and considered exploratory.

It is also important to note that this study relied on a single-site convenience sample and self-reported data, both of which limit the generalizability of the findings. These factors may also lead to an overestimation of the actual competency levels. Therefore, the results are best understood as specific to this context rather than reflective of all nursing students in Saudi Arabia. Nevertheless, this study offers useful baseline information that can support local curriculum improvements and inform future research, particularly larger multisite or mixed methods studies aimed at strengthening nursing informatics education in the country.

## Abbreviations

AI: artificial intelligence
C-NICAS: Canadian Nurse Informatics Competency Assessment Scale
EHR: electronic health record
ICT: information and communications technology
